# Essential Role for the d-Asb11 *cul5* Box Domain for Proper Notch Signaling and Neural Cell Fate Decisions *In Vivo*

**DOI:** 10.1371/journal.pone.0014023

**Published:** 2010-11-19

**Authors:** Maria A. Sartori da Silva, Jin-Ming Tee, Judith Paridaen, Anke Brouwers, Vincent Runtuwene, Danica Zivkovic, Sander H. Diks, Daniele Guardavaccaro, Maikel P. Peppelenbosch

**Affiliations:** 1 Hubrecht Institute-KNAW and University Medical Center Utrecht, Utrecht, The Netherlands; 2 Department of Cell Biology, University Medical Center Groningen, University of Groningen, Groningen, The Netherlands; 3 Department of Gastroenterology and Hepatology, Erasmus MC-University Medical Center Rotterdam, Rotterdam, The Netherlands; University Medical Center Maastricht, Netherlands

## Abstract

ECS (Elongin BC-Cul2/Cul5-SOCS-box protein) ubiquitin ligases recruit substrates to E2 ubiquitin-conjugating enzymes through a SOCS-box protein substrate receptor, an Elongin BC adaptor and a cullin (Cul2 or Cul5) scaffold which interacts with the RING protein. *In vitro* studies have shown that the conserved amino acid sequence of the cullin box in SOCS-box proteins is required for complex formation and function. However, the *in vivo* importance of cullin boxes has not been addressed. To explore the biological functions of the cullin box domain of ankyrin repeat and SOCS-box containing protein 11 (d-Asb11), a key mediator of canonical Delta-Notch signaling, we isolated a zebrafish mutant lacking the Cul5 box (Asb11^Cul^). We found that homozygous zebrafish mutants for this allele were defective in Notch signaling as indicated by the impaired expression of Notch target genes. Importantly, *asb11^Cul^* fish were not capable to degrade the Notch ligand DeltaA during embryogenesis, a process essential for the initiation of Notch signaling during neurogenesis. Accordingly, proper cell fate specification within the neurogenic regions of the zebrafish embryo was impaired. In addition, Asb11^Cul^ mRNA was defective in the ability to transactivate a *her4::gfp* reporter DNA when injected in embryos. Thus, our study reporting the generation and the characterization of a metazoan organism mutant in the conserved cullin binding domain of the SOCS-box demonstrates a hitherto unrecognized importance of the SOCS-box domain for the function of this class of cullin-RING ubiquitin ligases and establishes that the d-Asb11 cullin box is required for both canonical Notch signaling and proper neurogenesis.

## Introduction

**Figure 1:**
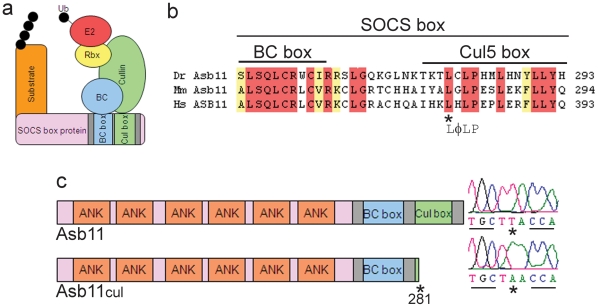
Schematic representation of Asb11 proteins. (**A**), Asb11 functions as a substrate recognition module in a putative Elongin BC-Cullin-SOCS-box (ECS) type E3 ubiquitin ligase complex. (**B**), Sequence alignment of conserved Asb11 SOCS-box domain in different species. The cul5-box consensus sequence is indicated below the alignment. Identical amino acids are highlighted in *red* and similar ones in *yellow*. Dr: *Danio rerio*; Mm: *Mus musculus*; Hs: *Homo sapiens*. (**C**), (*left*) Illustration of the wild type and mutant *d-asb11* gene products. Mutated protein is represented as Asb11^cul^ showing the predicted residual fragment and the position of the identified mutation. The different domains are indicated. (*right*) The T→A mutation changes a leucine into a stop codon.

The ubiquitin-proteasome system plays a fundamental role in the control of numerous cellular processes, including cell cycle progression, gene transcription, signal transduction, proliferation and differentiation [1]. In this system, ubiquitin is first activated by an E1 ubiquitin-activating enzyme. Activated ubiquitin is then transferred to the active-site cysteine of an E2 ubiquitin-conjugating enzyme. Subsequently, an E3 ubiquitin ligase mediates the transfer of ubiquitin from E2 to a lysine residue on the protein substrate. Multiple rounds of these reactions lead to the formation of polyubiquitylated substrates that are targeted to the 26S proteasome [2]. There are two major classes of E3 ubiquitin ligases, proteins with a HECT (homologous to E6-AP carboxyl terminus) domain and proteins with a RING (Really Interesting New Gene)–like motif. Within this class, cullin-RING E3s are multisubunit ubiquitin ligases composed of a scaffold protein known as cullin, a RING finger protein, which mediates the interaction with the E2, a variable substrate-recognition subunit and an adaptor that links the cullin-RING complex to the substrate recognition subunit [3]. Among the cullin-RING E3s, the group collectively denominated as ECS (Elongin BC-Cul2/Cul5-SOCS-box protein) ubiquitin ligases has recently attracted special attention [4]. This group of E3 ligases has been implicated in transduction of extracellular cues to altered gene transcription. Many details of its modus operandi remain, however, obscure. Specifically, there is remarkably little insight into the *in vivo* relevance of the different components of ECS ubiquitin ligases. *In vitro* studies have shown that in ECS ubiquitin ligases the SOCS-box protein works as the substrate recognition subunit. SOCS-box proteins are composed of two distinct protein-protein interaction domains, a substrate binding domain and a SOCS-box domain. The SOCS-box motif is found at the C-terminus of over 70 human proteins in nine different families. *In vitro* studies show that SOCS boxes act as substrate recognition modules of the ECS type E3 ubiquitin ligase complex (Fig. 1A) [2]. The SOCS-box domain is divided into two sub-domains: the BC box, which links SOCS-box proteins to the cullin-Rbx module and a motif termed cullin box, located immediately downstream of the BC box. The cullin box is proposed to determine whether a given SOCS-box protein assembles into either a Cul2-Rbx1 or a Cul5-Rbx2 module to recruit and activate the E2 ubiquitin-conjugating enzymes for substrates ubiquitylation [5]–[8]. *In vivo* evidence that the cullin box is involved in mediating the biological action of any SOCS-box protein has not been provided hitherto.

Ankyrin repeat and SOCS-box containing proteins (ASB) constitute the largest subclass of the SOCS-box protein family. ASB members (ASB1-ASB18) are structurally characterized by a variable number of N-terminal ankyrin repeats, which mediate the association with the substrate [9]. ASB proteins in general participate in various important biological processes [10]–[17], but like the superfamily of SOCS-box proteins *in toto*, their role *in vivo* remains largely unknown. We have recently showed that *Danio rerio* Asb11 (d-Asb11) regulates compartment size in the endodermal and neuronal lineages [10] via ubiquitylation of DeltaA, leading to the activation of the canonical Notch pathway [11]. Thus, d-Asb11 is an attractive protein to assess the elusive functions of the cullin box motif in the SOCS-box holodomain. All ASB proteins share, with slight divergences, the consensus sequences of BC box and Cul5 box in their C-terminal (Fig. 1B)[5], [6], [8]. Thus elucidation of the *in vivo* mode of action of d-Asb11 should also provide important clues for this family in its entirety. Together, these considerations prompted us to explore the function of Asb11 cullin box *in vivo*.

Here, we describe the isolation of a zebrafish carrying a mutant allele in the conserved LPφP sequence of the d-Asb11 cullin box. This mutant represents the first metazoan harboring a mutated cullin box. *asb11^Cul^* fish are defective in Notch signalling and have severely affected cell fate specification within the neurogenic regions of zebrafish embryos. Thus, our results establish a previously unrecognised *in vivo* importance of the cullin box for SOCS-box proteins in general and for Asb11 SOCS-box protein function in particular.

## Results and Discussion

### Generation and characterization of *d-asb11* mutants

The consensus sequence φ*XX*LPφPXXφX*X*(Y/F) corresponds to the Cul5-box in the C-terminal portion of the canonical SOCS-box proteins, and is highly conserved in vertebrates [5], [9] (Fig. 1B). We performed a TILLING screen on an F_1_ *N*-ethyl-*N*-nitrosurea (ENU)-mutagenized zebrafish library for *d-asb11* mutations mapping to the putative consensus sequence [18]. A premature stop codon corresponding to amino acid 281 in the conserved LPφP sequence of the d-Asb11 was identified (Fig. 1C), and the homozygous allele was designated *asb11^cul^*. To our knowledge, this is the first report of a metazoan mutant presenting a mutation in the consensus sequence of any SOCS-box protein, allowing for the first assessment of the *in vivo* function of the cullin box.

**Figure 2:**
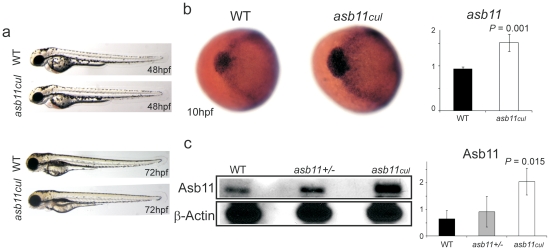
Phenotypic assays on wild type and *asb11^cul^* embryos. (**A**), Morphological analysis of wild type and mutant embryos at 48 and 72hpf. (**B**), (*left*) Anterior view of wild type and mutant embryos at 10hpf after whole mount *in situ* hybridization, WISH, using probe against *d-asb11*. (*right*) Graph shows the quantification of the respective expressions using qPCR. (**C**), (*left*) Endogenous d-Asb11 in wild type (WT), heterozygous (asb11+/−) and mutant (*asb11^cul^*) embryos at 12 hpf was detected by immunoblotting using anti-d-Asb11 antibody. (*right*) Graph quantifies 3 individual experiments, with 30 embryos/genotype/experiment.

Morphological analysis of *asb11* ^cul^ revealed a slight hyperpericardium at 48 and 72 hours post-fertilization (hpf) (Fig. 2A). This corresponds to the Asb11 knockdown morphant phenotype we described previously, although with less severity [10].

Next, to further identify the functional consequences of the mutated allele, we performed whole-mount *in situ* hybridization (WISH) with *d-asb11* probe on 10 hpf embryos. Strikingly, d-*asb11* transcripts were enhanced in *asb11^cul^* mutants compared to wild type, showing expanded expression in the polster, a U-shaped structure surrounds the head [19], and along the margins of the neural plate (Fig. 2B). Quantitative RT-PCR (qPCR), confirmed the increase of mRNA transcripts in *asb11* ^cul^. Accordingly, higher protein expression levels were detected by immunoblotting on 12 hpf lysates from *asb11^cul^* embryos (Fig. 2C). No significant quantitative differences between wild type and heterozygous embryos confirmed the recessive nature of the mutation. The higher mRNA transcripts and protein levels suggest a compensatory effect of a hypomorphic mutation in the *asb11^cul^* embryo (we can exclude that this works through reduced Notch signaling as DAPT treatment reduces Asb-11 and forced Notch signaling increases Asb-11 expression [11]), implying that the cullin box mutation has consequences for d-Asb11 function.

### Cullin box is required for correct expression of Notch target genes

**Figure 3:**
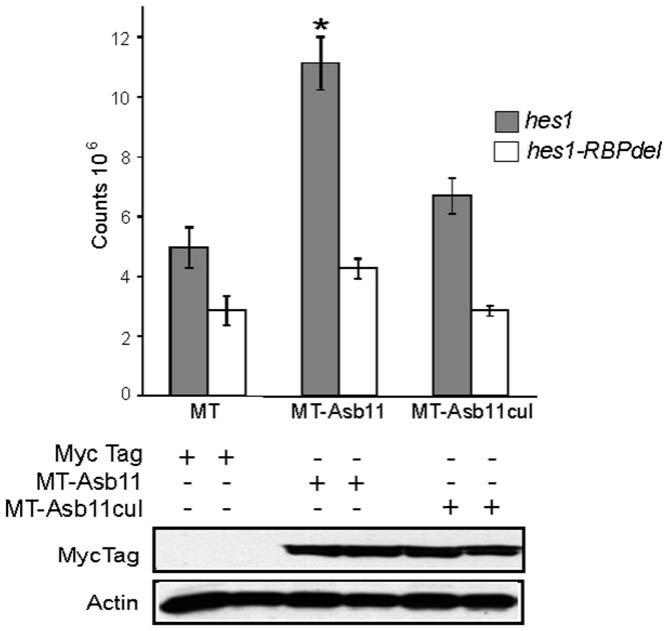
Cullin box domain promotes induction of *hes1* gene *in vitro*. nTera-d1 cells were co-transfected with *hes1-luciferase* (*hes1*) or *hes1-luciferase* lacking the conserved CSL-binding site (*hes1-RBPdel*) and *myc-tag* (MT) as a control, or myc-tagged *d-asb11* full length (MT-Asb11) or myc-tagged *asb11^cul^* (MT-Asb11^cul^) cDNA. Hes1-dependent Notch activity was analyzed by luciferase measurement.

Morpholino-mediated knockdown of *d-asb11* causes repression of specific Delta-Notch elements and their transcriptional targets, whereas misexpression of *d-asb11* induces Delta-Notch activity [11]. To test whether the cullin box mutation has comparable consequences for d-Asb11 function in regulating Delta-Notch signaling pathway, we first explored the capacity of the cullin box-deleted protein to activate, upon its overexpression, Notch-dependent transcription *in vitro*. We observed that overexpression of wild type d-Asb11 in human neuronal precursor cell line, NTera2 [20] led to a strong activation of the Notch target gene *hes1*, however, overexpression of the mutant protein was not capable of doing so (Fig. 3). Because Notch signaling induces activation of *hes1* gene through the CSL transcriptional complex [21], we used a *hes1* reporter lacking the conserved CSL-binding site (*hes1-RBP*) to confirm Notch-specificity for this transactivation. However, neither d-Asb11 nor Asb11^Cul^ were capable of transactivating *hes1-RBP*. These results showed that the Cul5 box of d-Asb11 is essential for its function to activate the Notch target gene *hes1* through Notch pathway.

**Figure 4:**
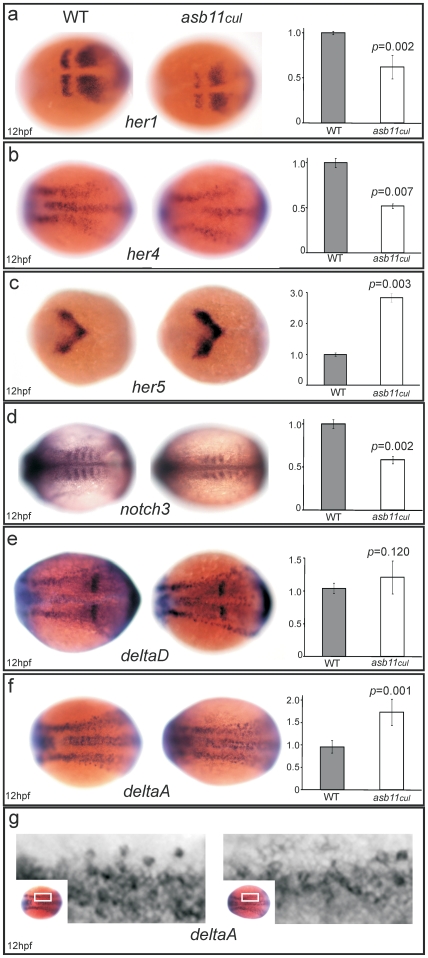
*asb11^cul^* presented altered expression of Delta-Notch pathway components. Wild type (*left panel*) and mutant (*middle panel*) embryos at 12 hpf were analyzed for WISH using probes against *her1*, **A**; *her4*, **B**; *her5*, **C**; *notch3*, **D**; *deltaD*, **E**; *and deltaA*, **F**. (**G**), Higher magnification shows detailed analysis of *deltaA* expression. (*left*) Graphs quantify the mRNA expression levels.

Next, we investigated the expression of Notch target genes *in vivo* by performing WISH for the Hairy/E(spl)-related transcription factors, *her1*, *her4* and *her5* on 12 hpf *asb11^cul^* and wild type embryos. At this time point, the expression of *her1* and *her4* was considerably reduced in *asb11^cul^* embryos (Fig. 4A–B). As *her1* and *her4* are known to be activated by the Notch signaling [22], this result suggests that the Notch signaling pathway is disrupted in embryos lacking the cullin box domain of Asb11. In contrast, asb11^cul^ showed a significant increase in the expression of *her5* (Fig. 4C), which is known to be downregulated by the Notch1A-intracellular domain [23]. Consistently, we observed downregulation of *notch3* (Fig. 4D), which has been shown to repress *hes5*, a mammalian homologue of zebrafish *her5* [24] (although Notch inhibition does not expand the *her5* expression domain *per se* [25], and thus the exact status of *her5* as a Notch target gene remains uncertain). The other Notch genes have not been reported to change expression of *her5* at this stage of zebrafish embryogenesis, thus we did not attempt to assess their expression levels in the context of the analysis of *her5* expression patterns. Next, we analyzed expression of the Notch ligands DeltaA and DeltaD in *asb11^cul^* embryos. *deltaA* transcripts showed increased expression in *asb11^cul^* embryos (Fig. 4F), whereas *deltaD* remained unaffected (Fig. 4E). Detailed examination of the WISH expression patterns of *deltaA* revealed a change in distribution of mRNA in the neural plate (Fig. 4G). Wild type embryos exhibit a distinct “salt and pepper” aspect of *deltaA* mRNA distribution whereby some cells have stronger expression than their neighbors, consistent with the notion of Delta-Notch lateral signaling [26]. In contrast, *asb11^cul^* embryos showed a smear of *deltaA* mRNA transcript across the neural plate, indicating an impaired Notch-mediated lateral inhibition. Thus, the mutation in the *d-asb11* cullin box results in the disruption of canonical Delta-Notch signaling.

### The cullin box domain of Asb11 is a bona-fide promoter of Notch-mediated *her4* induction expression

**Figure 5:**
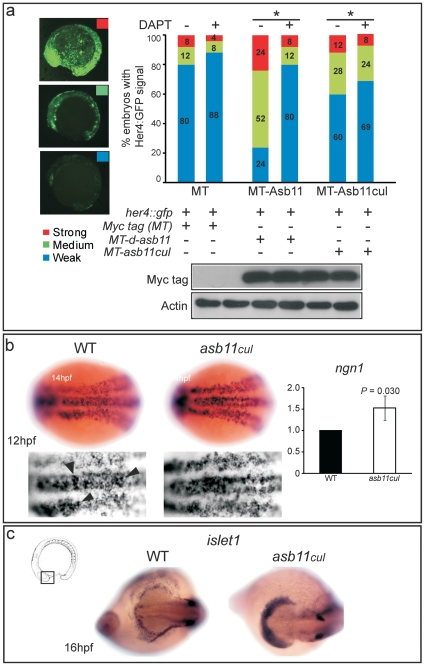
*her4::gfp* transactivation and premature differentiation of neural cells in *asb11^cul^*. (**A**), the *her4::gfp* reporter was co-injected with *myc-tag* (MT) mRNA as a control, myc-tagged *d-asb11* full length (MT-Asb11) or myc-tagged *asb11^cul^* (MT-Asb11^cul^) mRNA in zebrafish embryos. Injected embryos were treated with (+) (n = 25) or without (−) (n = 25) DAPT, from 1.5 hpf. At 14 hpf, embryos were analyzed for *her4* transactivation based on the intensity of the GFP signal. Positive embryos were counted and percentages of embryos presenting weak (blue), medium (green) or strong (red) signal were given. (**B**), Wild type (*left panel*) and mutant (*middle panel*) embryos at 12 hpf were analyzed for WISH using probe against *ngn1*. (*right*) Graph quantifies expression of *ngn1* using qPCR. (**C**) Wild type (*left panel*) and mutant (*right panel*) polster of embryos at 16 hpf were analyzed for WISH using probe against *islet1*.

It was reported that Hairy/E(Spl) expression and activity can be independent of Notch signaling *in vivo* [27]. Hence, to determine whether the altered regulation of Hairy/E(spl)-related transcription factors in *asb11^cul^* embryos was mediated by Notch activity, we co-injected *her4::gfp* reporter DNA with *d-asb11* or *asb11^cul^* mRNA in zebrafish embryos, which were then treated with DAPT, a γ-secretase inhibitor that blocks Notch signaling [28]. *her4* transactivation was determined as a summation of all green fluorescent protein (GFP) present in the embryo. Confocal microscopy was used to trinomially classify transactivation of the *her4* promoter as weak, medium or strong (Fig. 5A). When *her4::gfp* was injected with *myc tag* (*MT*) mRNA as a control, embryos presented 80%, 12% and 8% of weak, medium and strong GFP signals, respectively. Upon DAPT treatment, the number of medium and strong signal expressing embryos decreased to 8% and 4%, respectively, showing that Notch signaling was disrupted in response to DAPT treatment. Misexpression of *MT-d-asb11* mRNA resulted in an increase in embryos expressing medium GFP signals (52%, c.f. 12% in *MT*-injected embryos; p<0.05), and strong GFP signals (24% c.f. 8% in *MT*-injected embryos; p<0.05). In agreement with previous data, MT-dAsb11 was unable to induce *her4:gfp* upon exposure of DAPT [11], showing the hierarchical upstream function of d-Asb11 in canonical Notch activation.

Interestingly, injection of MT-Asb11^cul^ mRNA caused an increase in the number of embryos expressing medium signals, whereas the number of embryos with strong *her4::gfp* expression was slightly increased compared with control *MT*-injected embryos. However this effect was observed in both DAPT treated and untreated embryos (24% and 28%, respectively), suggesting that d-Asb11 lacking the cullin box domain (Asb11^cul^) is much less efficient in inducing the *her4* reporter than wild type d-Asb11 and its function is independent of Notch signaling. These data are consistent with studies showing that *her4* may be expressed in a Notch-independent manner in specific regions of the nervous system [27]. Although during early neurogenesis *her4* expression requires Notch activation, during late neuronal development the *her4* induction in sensory neurons is independent of Notch signaling and dependent on proneural genes, as *neurogenin1* (*ngn1*) and *zath3* [29].

We performed WISH to investigate the expression of *ngn1*, a bHLH transcription factor, which is expressed in neuronal precursors and differentiated neural cells [30] and is negatively regulated by Notch signaling [31]. As expected, wild type embryos at 12 hpf displayed the typical clustered expression of *ngn1* (Fig. 5B). However, *asb11^cul^* embryos expressed *ngn1* at a uniform high level with less evidence of clustering. The increase in *ngn1* mRNA expression was confirmed by qPCR. d-Asb11 morphants showed a similar phenotype [10], confirming that the higher expression of *ngn1* is caused by loss of d-Asb11 function in the mutant.

Some studies have shown that *her4* is also expressed in Islet1/2- positive sensory neurons and its expression is not involved in canonical Notch signaling [29]. Consistently, *islet1*, detected by WISH, was also increased in zebrafish mutants at 16 hpf. Interestingly, *islet1* expression was higher in the polster region where *asb11^cul^* were significantly increased in mutants (Fig. 5C).

All together our data suggest that the cullin box domain of d-Asb11 is essential to regulate Notch targets genes although d-Asb11 lacking the cullin box may yet affect protein expression independently of Notch, via proneural genes.

### The cullin box is essential for DeltaA degradation and regulation of neural committed cells

**Figure 6:**
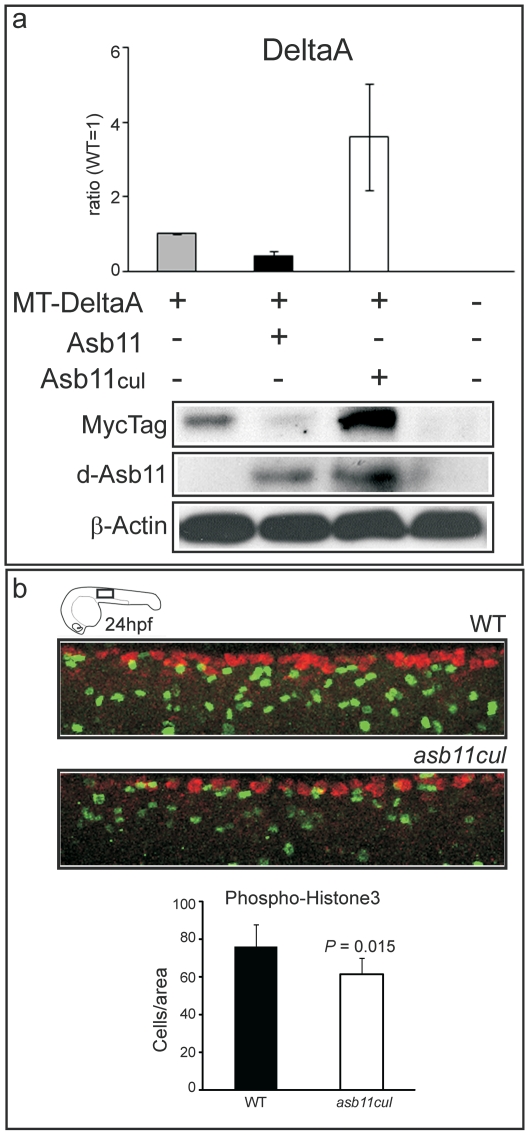
Cullin box is essential for DeltaA degradation and for maintaining a cell proliferating state *in vivo*. (**A**) Zebrafish embryos were injected with Myc-tagged *deltaA* (MT-DeltaA) and *d-asb11* (Asb11) or *asb11^cul^* (Asb11^cul^) mRNA at one-cell stage. (*lower panel)* Lysates of 12 hpf embryos were analyzed by immunoblotting for the presence of DeltaA. (higher panel) Graph quantifies 2 individual experiments, each with 30 injected embryos/group. (**B**), Fluorescent whole-mount antibody labeling of wild type (WT) and *asb11^cul^* embryos at 24 hpf for the mitotic marker anti-phosphohistone-3 (PH 3) antibody (*green*) and the neuronal marker Hu(C). Graph shows the number of positive cells per area (5 somites from beginning of yolk extension) of 5 embryos for each genotype.

We have previously shown that d-Asb11 affects Delta-Notch signaling by targeting DeltaA for ubiquitylation and subsequent degradation. This effect, strictly dependent on the presence of the SOCS-box [11], establishes the lateral inhibition gradients between DeltaA and Notch facilitating canonical Notch signaling. To study the role of the cullin box domain in d-Asb11-mediated degradation of DeltaA, we injected zebrafish embryos with Myc-tagged *deltaA* (MT-*dlA*) and *d-asb11* or *asb11^cul^* mRNA at one-cell stage. Embryos were analyzed for the presence of MT-DeltaA protein at 12 hpf. Wild type embryos injected with full-length *d-asb11* displayed substantial DeltaA degradation. In contrast, injected *asb11^cul^* was not capable of degrading DeltaA when compared to control (Fig. 6A; p<0.05).

Thus, we show that the cullin box domain of d-Asb11 is essential for degradation of Notch ligand DeltaA in zebrafish embryos, providing the first *in vivo* (but not in vitro, *e.g.* [32]]) evidence that absence of a cullin box interferes with a protein degradation function of a SOCS-box- protein. Moreover, the expression of *deltaA* in the three longitudinal domains of zebrafish neural plate corresponds to regions that express elevated levels of *ngn1* and in which the earliest neurons are born [33]. As Asb11^cul^ was unable to degrade DeltaA and acted as a dominant negative increasing the quantity of DeltaA protein in mutant embryos, we propose that the premature neuronal commitment in *asb11^cul^* embryos, assessed by the increased expression of the proneural gene *ngn1*, is a consequence of DeltaA accumulation in the neural plate.

### Absence of the cullin box alters proliferation patterns

As Notch signaling drives (or maintain) precursor cell proliferation within the neurogenic regions of the embryo, a prediction from our findings would be that the loss of d-Asb11 cullin box would impair such proliferation. Indeed, fluorescent whole-mount antibody labeling with the mitotic marker anti-phosphohistone-3 (PH 3) antibody showed a significant decrease in the rate of cellular proliferation of *asb11^cul^* embryos at 24 hpf (Fig. 6B, *green label*), indicating that the d-Asb11 cullin box is necessary for proper cell proliferation. Alternatively, the premature differentiation of precursor cells in *d-asb11* mutants led to diminished number of proliferating cells.

In summary, here we show that the Cul5 domain of d-Asb11 is necessary for proper Notch signaling *in vitro* and *in vivo*. Zebrafish embryos lacking the cullin box of d-Asb11 displayed alterations in the expression of Notch pathway components and defective neurogenesis. Thus, our *in vivo* study reveals a novel role of cullin boxes previously unrecognized in *in vitro* experiments.

## Materials and Methods

### Fish and embryos

Zebrafish were kept at 27.5°C. Embryos were obtained by natural matings, cultured in embryo medium and staged according to methods previously described [34].

### Plasmid construction

Plasmids were constructed and/or provided as previously described [10], [11]. The pCS2^+^MT-DeltaA construct was provided by B. Appel (Vanderbilt University, Nashville TN) [35]. The *her4::gfp* reporter was provided by S. Yeo (Kyungpook National University, Korea) [2]. For *asb11^cul^*, mutant zebrafish cDNA was isolated and cloned into the EcoRI and XhoI sites of pCS2^+^MT and pCS2^+^.

### mRNA synthesis, mRNA and DNA microinjections

Capped mRNAs were synthesized using the mMESSAGE mMACHINE kit (Ambion). Fig. 6A, embryos were injected with 600 pg *MT-deltaA* and 350 pg *d-asb11* or 350 pg *asb11^cul^* mRNAs. Fig. 5A, embryos were injected with 5 pg *her4::gfp* DNA or 5 pg *her4::gfp* +300 pg *d-asb11* or *asb11^cul^* mRNA. Total volume of the injection was set at 1 nL.

### DAPT treatment

Half of each injected group (n = 50) (Fig. 5A) was incubated in 100 µM DAPT diluted in 1% DMSO in embryo-medium (5 mM NaCl, 0.17 mM KCl, 0.33 mM CaCl_2_, 0.33 mM MgSO_4_, 0.00005% Meth Blue). The other half was incubated in 1% DMSO in embryo-medium. The embryos were incubated from 1.5hpf till 14hpf, fixed with 4% PFA overnight at 4°C and analyzed for GFP expression.

### *In situ* hybridization

Whole mount *in situ* hybridizations were performed according to methods previously described [36]. All probes used in this study are previously described [10], [11].

### Immunoblotting

Whole mount *in situ* hybridizations were performed according to methods previously described [36]. At 12hfp, chorion and yolk were removed. Embryos were lysed in cell lyses buffer (50 mM Tris-Cl pH 7.5, 150 mM NaCl, 1 mM EDTA, 0.1% Na-deoxycholate, 1% NP-40, 10 u, 1% protease inhibitor (ROCHE), 2 µl/embryo. Primary antibodies were diluted in PBS containing 5% milk (fig. 2: rabbit anti-asb11 1∶100, fig. 5: rabbit anti-MT 1∶1000, Bioke) and used for immunoblotting as previously described [37]. As loading control an anti-actin body was used in addition to coomassie staining of the membrane. For densitometric analysis all bands were measured with a GS-800 Densitometer (Biorad), and total area counts (OD x mm^2^) were corrected for back ground (equivalent area on a non-relevant place on the blot). Subsequently samples were corrected for loading using the control band and finally values were expressed relative, defining the intensity of the wild type sample as 1.

### RNA isolation and qRT-PCR

Total RNA was extracted from whole wild type and mutant embryos at 10 or 12 hpf. Total RNA extraction, cDNA synthesis and qPCR quantification were performed according to previously described methods [38].

### Whole mount immunolabelling, microscopy and image quantification

Whole-mount immunohistochemistry and picture capture and analysis was performed as described [13], [39]. For figure 6B anti-HuC (red) and anti-PH 3 (green) antibodies (Upstate Biotechnology) were used. For the analysis of fluorescent stainings, Leica Confocal TCS SPE was used. To quantify the intensity of signal, a z-stack (z-slices of 7 µM) was made, scanning the whole embryo. Leica software (Application Suite 1.8.0) was used to create a maximum projection of the z-stack.

### Luciferase reporter assay

nTera2/d1 cells were maintained in DMEM containing 10% FCS. The culture media were supplemented with 5 mM glutamine and antibiotics/antimycotics. Cells were incubated at 5% CO_2_ in a humidified incubator at 37°C. NTera2/d1 cells were seeded in a 96-well plate and transfected using IBAfect and MA-enhancer (IBA Biosciences, GmbH) using the suppliers protocol. Luciferase was measured on a Packard TOPCOUNT Microplate Scintillation Counter (Packard). The experiments were performed two times in triplicate. Values were normalised with TAL-luc [11].

### Statistical testing

Each value with a standard deviation is the average of at least two independent experiments performed in triplicate. Statistical tests were performed using two-tailed t-test. All bars in graphs depict mean values with error bars depicting standard deviations. Statistical χ^2^-test was performed for Fig. 5A.
